# Healing of Fatigue Crack by High-Density Electropulsing in Austenitic Stainless Steel Treated with the Surface-Activated Pre-Coating

**DOI:** 10.3390/ma6094213

**Published:** 2013-09-23

**Authors:** Atsushi Hosoi, Tomoya Kishi, Yang Ju

**Affiliations:** Department of Mechanical Science and Engineering, Nagoya University, Furo-cho, Chikusa-ku, Nagoya 464-8603, Japan; E-Mails: hosoi@mech.nagoya-u.ac.jp (A.H.); kishi.tomoya@e.mbox.nagoya-u.ac.jp (T.K.)

**Keywords:** healing, crack, fatigue, electropulsing, propagation, coating

## Abstract

A technique to heal a fatigue crack in austenitic stainless steel SUS316 by applying a controlled, high-density pulsed current was developed. A surface-activated pre-coating (SAPC), which eliminates the oxide layer and coats a Ni film on the crack surface, was used to improve the adhesion between crack surfaces. Cracks were observed by scanning electron microscopy before and after the application of high-density electropulsing. To evaluate the healing effect of the SAPC during crack propagation, fatigue tests were conducted under a constant stress intensity factor. The fatigue crack treated with the SAPC was found to be effectively healed as a result of electropulsing, and also showed a slower rate of crack propagation.

## 1. Introduction

Fatigue is the main cause of failures in metallic structures. Various techniques have been developed to improve fatigue strength, such as high-frequency quenching, carburizing, nitriding, shot peening, and laser shock peening. Implementation of these techniques can prolong the fatigue life of materials because crack initiation is suppressed by hardening and residual compressive stress on the material surface [1,2,3,4]. However, the effectiveness of these methods is limited to materials where a crack already exists. Thus, it is important to develop a crack healing technique to improve the long-term reliability and durability of metallic structures with existing cracks.

Recently, there have been several studies regarding to crack healing techniques. For polymer materials, White* et al.* [5] reported a method that uses microencapsulation and Chen* et al.* [6] proposed a method based on thermal reversibility. For ceramic materials, a technique to heal surface cracks by oxidation was reported [7]. For metallic materials, a healing technique using precipitation was proposed by Shinya* et al.* [8], who succeeded in healing creep cavities by promoting the precipitation of boron nitride on an austenitic stainless steel modified with boron, cerium, and titanium. Lumley [9] showed that creep resistance can be enhanced and that fatigue cracks can be healed by dynamic precipitation in aluminum alloys. However, these healing techniques for metallic materials cannot be used for pre-existing structures because the precipitation elements must be added to the material in advance.

In this study, the application of high-density electropulsing is considered for the healing of cracks propagating in a pre-existing material. Certain effects of the application of high-density electropulsing on metallic materials have previously been studied, and it has been reported that high-rate heating, thermal stress, and electron collision during electropulsing cause certain phenomena, such as electroplasticity [10,11], recrystallization [12,13], phase transformation [14,15,16], electromigration [17] and dislocation motion [13,14,15,16,18]. Song* et al.* [19] and Zhu* et al.* [20] showed a recovery after plastic deformation owing to local recrystallization, phase transformation, and dislocation annihilation with high-density electropulsing. Conrad* et al.* [21], Sosnin* et al.* [22], and Konovalov* et al.* [23] showed that fatigue life is prolonged owing to the homogenization of slips on the material surface and the change of dislocation structures, where dislocation motion is caused by the electromigration effect. In addition, Suhong* et al.* [24] showed that persistent slip bands on the material surfaces vanished locally by high-density electropulsing. These studies are based on healing microscopic damage in metallic materials, but are not valid for cracks propagating in these materials. Zhou* et al.* [25,26] showed that a pre-crack introduced in medium carbon steel was closed and healed locally by heating and thermal compressive stress caused by high-density electropulsing. However, the effect of healing on the mechanical properties of the material has not been studied. We have previously developed a technique using closely-spaced electrodes to heal a fatigue crack introduced in austenitic stainless steel by high-density electropulsing [27]. The effect of healing on fatigue crack propagation was evaluated quantitatively and it was revealed that the crack was closed and bridging by partial melting occurred between crack surfaces. However, adhesion between crack surfaces was prevented owing to the presence of the oxide layer, and the effect of healing on crack propagation was only temporary.

In this study, we improved the adhesion of crack surfaces by applying a surface-activated pre-coating (SAPC), which eliminates the oxide layer and coats a Ni film on the crack surfaces. In addition, we quantitatively evaluated the healing of fatigue cracks treated with the SAPC by high-density electropulsing.

## 2. Experimental Details

### 2.1. Samples

**Figure 1:**
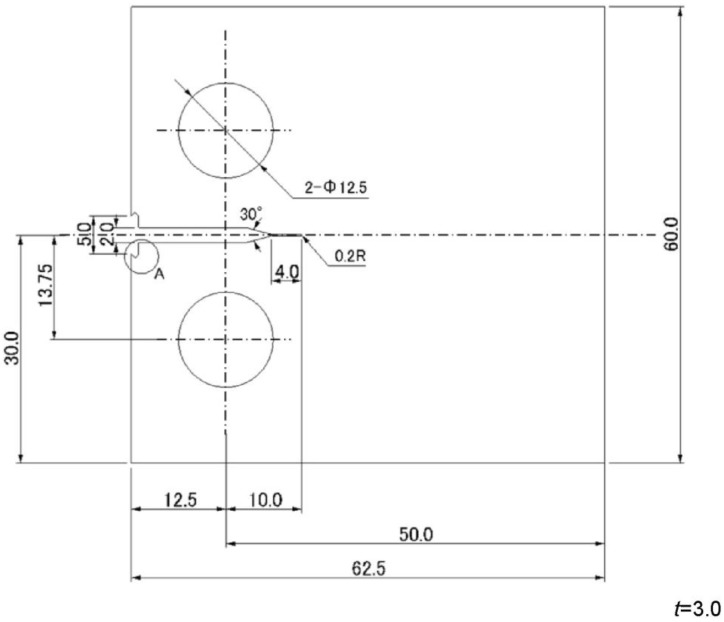
Schematic representation of the steel samples.

Austenitic stainless steel SUS316 was used as the experimental material. The chemical composition of the stainless steel SUS316 is C, Si, Mn, P, S, Ni, Cr, Mo and Fe, and their content rates are 0.05, 0.26, 1.3, 0.028, 0.03, 10.1, 17.09, 2.01 wt % and balance, respectively. The mechanical properties of the stainless steel SUS316, yield stress, tensile strength, Young’s modulus, Poisson’s ratio and hardness, are 300 MPa, 573 MPa, 193 GPa, 0.3 and 161 HBW, respectively. Compact tension (CT) samples were used and a schematic of the sample is provided in Figure 1. The samples were subjected to stress relief annealing to remove the residual stress induced during the machining process. The annealing process is described as follows. The samples were heated to 1173 K over a period 4 h. The temperature was then held constant at 1173 K for 10 min before the samples were allowed to cool to room temperature in the furnace. To facilitate the observation of the surface condition, the surfaces of the samples were polished to a mirror plane using a buffing machine.

### 2.2. Experimental Conditions

#### 2.2.1. Fatigue Test Conditions

Tensile fatigue tests were conducted to introduce a fatigue crack in the annealed sample and to quantitatively evaluate crack growth rate before and after healing. Tensile fatigue tests were carried out at room temperature in air with a hydraulically-driven testing machine. All tests were conducted at a stress ratio of 0.05 and a frequency of 10 Hz. The crack length under cyclic loading was measured from the compliance using a clip gauge. The details of the fatigue test conditions are shown in Table 1. The fatigue test was carried out for three kinds of samples (designated Sample A, B, and C) with controlled stress intensity factor. The crack healing process was observed in Sample A, whereas Sample B and C were used to quantitatively evaluate the healing effect by measuring the crack growth rate. For comparison, the crack growth rate was also measured on a control sample without application of high-density electropulsing. The control sample was tested by load control within the stress intensity factor range of 15–35 MPa·m^1/2^.

**Table 1:** Conditions of fatigue test.

Sample name	Sample A	Sample B	Sample C	Control
Stress ratio *R*	0.05	0.05	0.05	0.05
Frequency *f* [Hz]	10	10	10	10
Pre-crack length *a* [mm]	3.0	6.0	8.0	–
Stress intensity factor range ∆*K* [MPa·m^1/2^]	35	15	25	15–35

#### 2.2.2. Surface-Activated Pre-Coating (SAPC)

**Figure 2:**
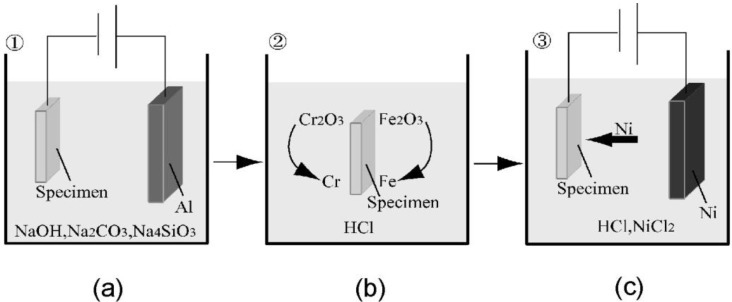
Schematic diagram of the three stages involved in the surface-activated pre-coating (SAPC): (**a**) electrolytic cleaning; (**b**) HCl activation; and (**c**) Ni striking.

The samples were subjected to the SAPC [28] to eliminate the oxide layer on the crack surfaces. The SAPC comprises three stages: electrolytic cleaning, HCl activation, and Ni striking. A schematic representation of this process is provided in Figure 2.

The samples were washed in pure water between each stage. During electrolytic cleaning, samples were washed in alkaline solution; the composition of the alkaline solution was 30 g/L NaOH, 30 g/L Na_2_CO_3_, and 30 g/L Na_4_SiO_3_. The anode was connected to an Al plate and the cathode was connected to the sample. The electrolytic cleaning current density was 10 A/dm^2^, the application duration of current was 60 s, and the temperature of the alkaline solution was kept at 333 K. During HCl activation, the oxide layer on the sample was eliminated after exposure to the 37% HCl solutions for 10 s. Finally, during Ni striking, the oxide layer on the sample was eliminated and the Ni film was coated, thereby preventing reoxidation. The composition of the Ni coating solution was 240 g/L NiCl_2_ and 80 g/L 37% HCl. The anode was connected to a Ni plate and the cathode was connected to the sample, and both were placed in the coating solution. The current density was 10 A/dm^2^ and the application duration of current was 90 s. Samples A and C were subjected to the SAPC, whereas Sample B was left untreated as a reference to monitor the effect of the SAPC on crack healing.

#### 2.2.3. Electropulsing Conditions

**Figure 3:**
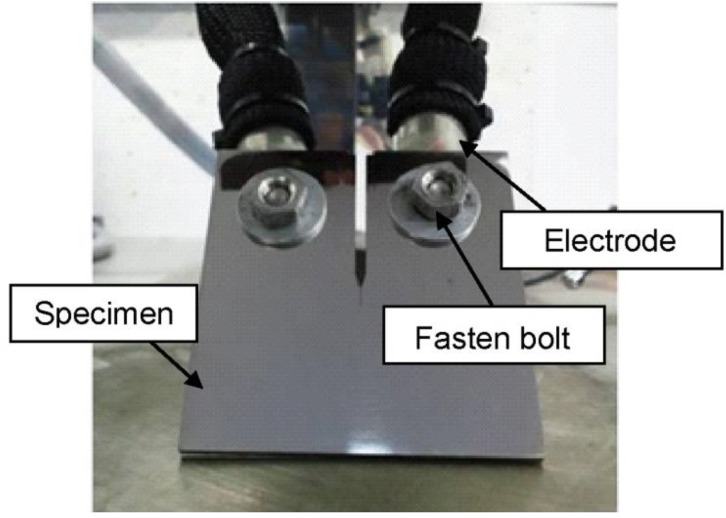
Photograph of the electropulsing set-up.

High-density electropulsing was applied to heal the fatigue crack. Electropulsing was carried out with a transistor-type power source in the range of 0.5–10 kA. The electropulsing was applied through the two electrodes with a pulse duration in the range of 0.5–10 ms. The electrodes were directly bolted to the sample as shown in Figure 3. The electropulsing conditions are shown in Table 2. Electropulsing was applied to the sample several times, and the state of the crack on the sample surface was observed with a scanning electron microscope (SEM) before and after the several applications of electropulsing.

**Table 2:** Conditions of electric current application.

Sample name	Sample A	Sample B	Sample C
Applied current [kA]	9.0	6.0	8.0
Pulse duration [msec]	2.0	2.0	4.0
Number of current application	35	25	20

## 3. Experimental Section

### 3.1. SAPC

**Figure 4:**
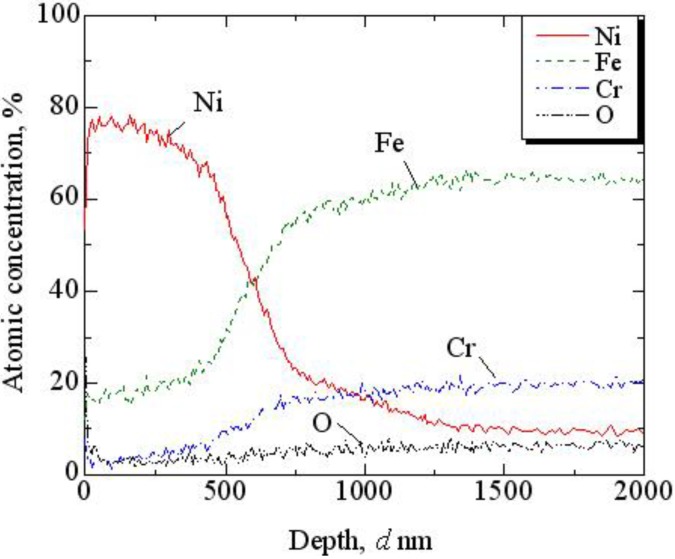
Relationship between the atomic concentrations of O, Cr, Fe and Ni and the depth from the sample surface after Ni striking.

Elemental analysis was performed with an Auger microprobe to investigate the effect of using the SAPC. Figure 4 shows the relationship between the atomic concentrations of O, Cr, Fe, and Ni and the depth from the sample surface after Ni striking. Fe and Cr are the main elements in the SUS316 sample, and Ni is the element coated by the SAPC. It is observed that the oxide layer is almost non-existent at the surface and the Ni coating is approximately 630 nm thick.

### 3.2. Fatigue Crack Observation

**Figure 5:**
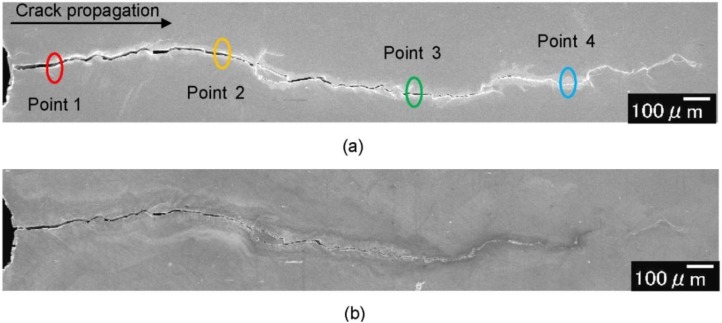

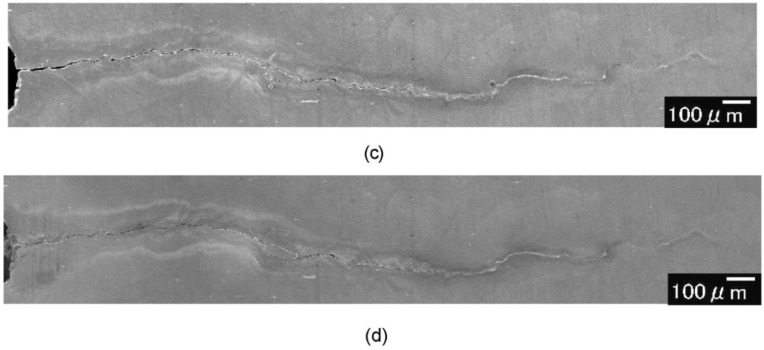
Fatigue crack closure in Sample A induced by high-density electropulsing: (**a**) before; (**b**) after 8; (**c**) after 25; and (**d**) after 35 applications of electropulsing.

**Figure 6:**
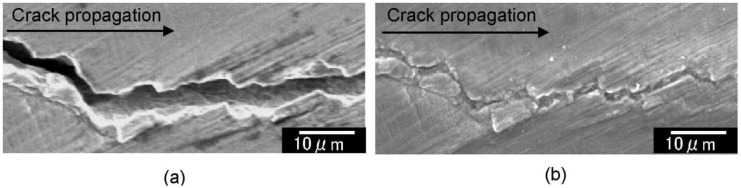
Magnified images of fatigue cracks in Figure 5: (**a**) before and (**b**) after 35 applications of electropulsing.

**Figure 7:**
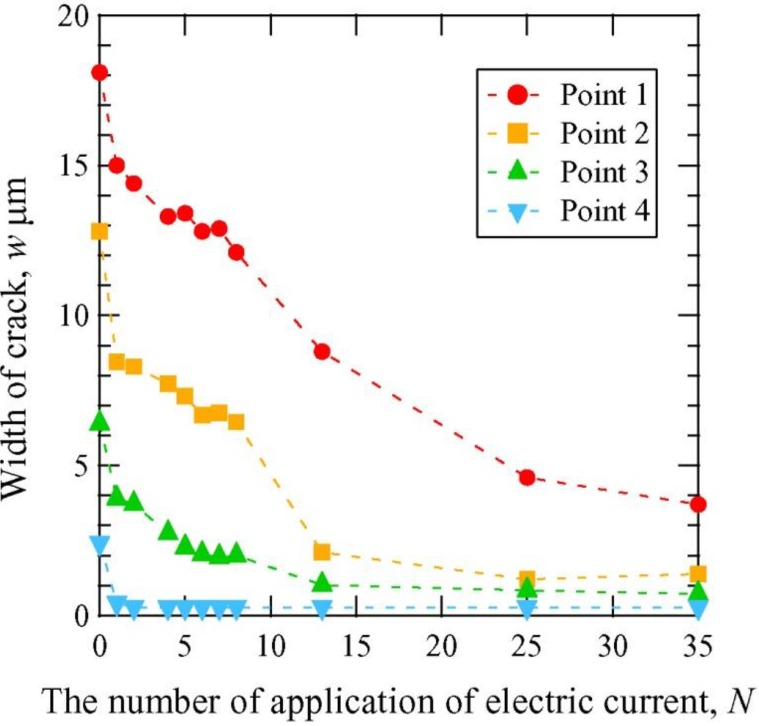
Crack width at the measuring point in Figure 5 as a function of the number of applications of electropulsing in Sample A.

Figure 5 shows SEM images of the crack closure in Sample A for different numbers of applications of high-density electropulsing. It is observed that the crack was closed at all stages. An enlarged image is shown in Figure 6 for an area approximately 1.2 mm from the crack tip before electropulsing and after the 35th application. Adhesion between the crack surfaces was observed and it was also noted that electropulsing affected the regions of the sample far from the crack tip. The crack width was measured from the SEM images before and after the application of electropulsing. Figure 7 shows the change in crack width with increasing number of electropulsing applications. Comparing the width before electropulsing and after the 35th application, the crack width near the notch decreased from 18.1 to 3.7 μm approximately, corresponding to a 79%–89% closure. The crack width after electropulsing was less than 5.0 μm. The bonding of the crack surfaces was confirmed by cutting the sample vertically in the direction of crack propagation.

### 3.3. Evaluation of Fatigue Crack Growth

**Figure 8:**
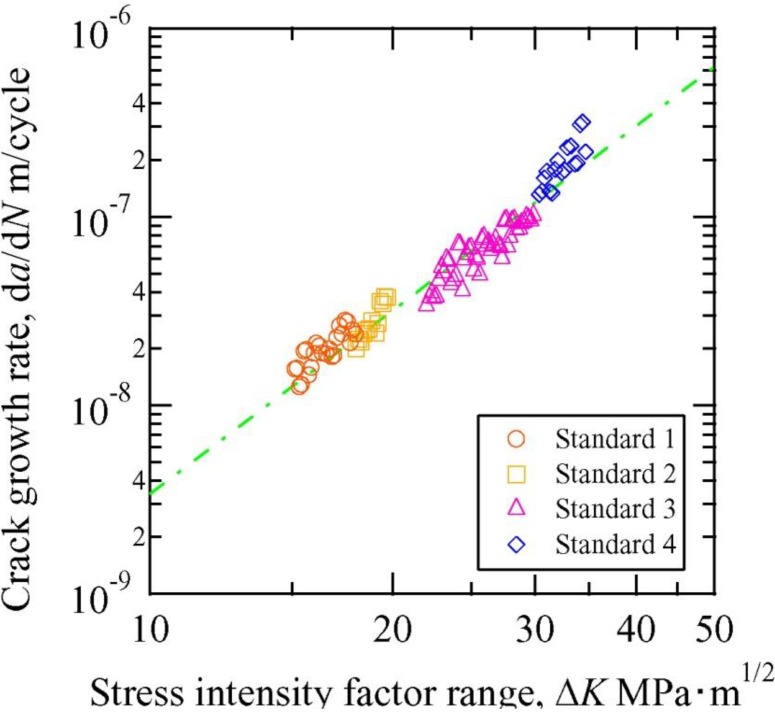
Fatigue crack growth rate as a function of stress intensity range for CT control samples without the application of electropulsing.

**Figure 9:**
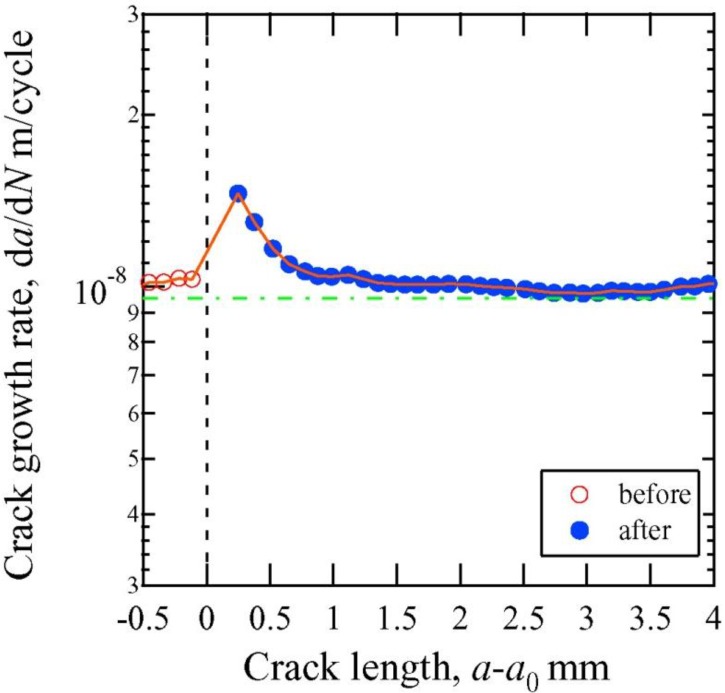
Fatigue crack growth rate as a function of crack length before the application and after the 25th application of electropulsing for Sample B.

**Figure 10:**
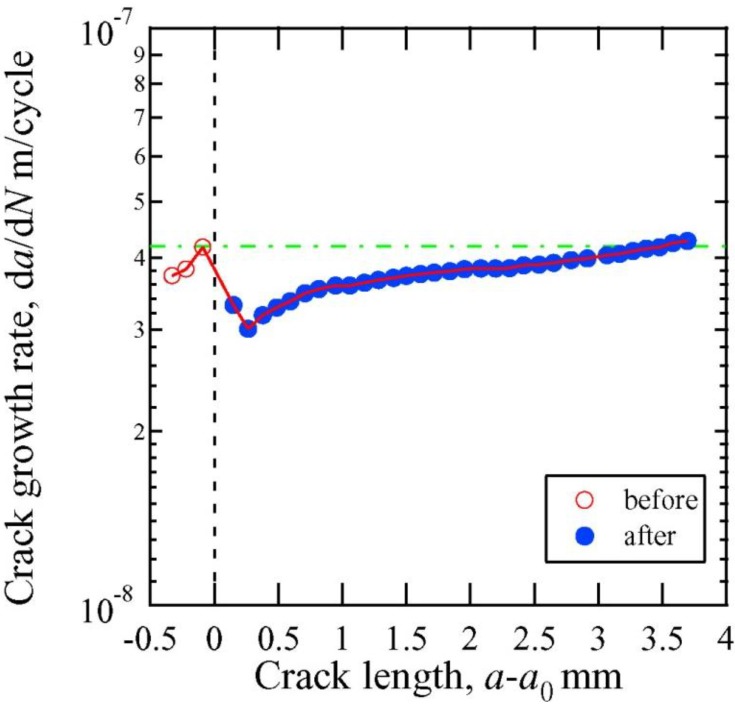
Fatigue crack growth rate as a function of crack length before the application and after the 20th application of electropulsing for Sample C.

The growth of the fatigue crack was evaluated quantitatively in Samples B and C to determine the effectiveness of crack healing. Crack closure was confirmed in Samples B and C, as was observed in Sample A. Figure 8 shows the crack growth rate as a function of the stress intensity factor (*i.e.*, Paris’ law) for the control sample in the absence of electropulsing. Figure 9 and Figure 10 show the relationship between the crack growth rate and crack length before and after electropulsing application in Samples B and C, respectively. In Figure 9 and Figure 10, *a*_0_ is the crack length before the application of electropulsing. The dashed line indicates the approximate value for the control sample when the fatigue test was carried out in the absence of electropusling. The open and solid symbols represent crack growth before and after electropulsing application, respectively.

Figure 9 shows the crack growth rate of Sample B, which was not subjected to the SAPC, before and after the 25th application of electropulsing. As indicated by the dashed line, the crack growth rate became constant when the fatigue test was conducted under a constant stress intensity factor (Δ*K* = 15 MPa·m^1/2^). However, it was observed that the crack growth rate increased from 1.03 × 10^−8^ to 1.46 × 10^−8^ m/cycle immediately after the application of electropulsing. Subsequently, the crack growth rate gradually decreased and when the crack length reached 1.4 mm, the growth rate returned to its initial value before the application of electropulsing.

Figure 10 shows the crack growth rate for Sample C, which was treated with the SAPC, before and after the 20th application of electropulsing. The fatigue test was conducted at a constant stress intensity factor (Δ*K* = 25 MPa·m^1/2^). From Figure 10, it was observed that the crack growth rate decreased from 4.17 × 10^−8^ to 3.01 × 10^−8^ m/cycle immediately after the application of electropulsing. After the application of electropusling, the crack growth rate was lower than that before electropulsing application until the crack had grown to 3.6 mm.

## 4. Discussions

### 4.1. Fatigue Crack Closure

**Figure 11:**
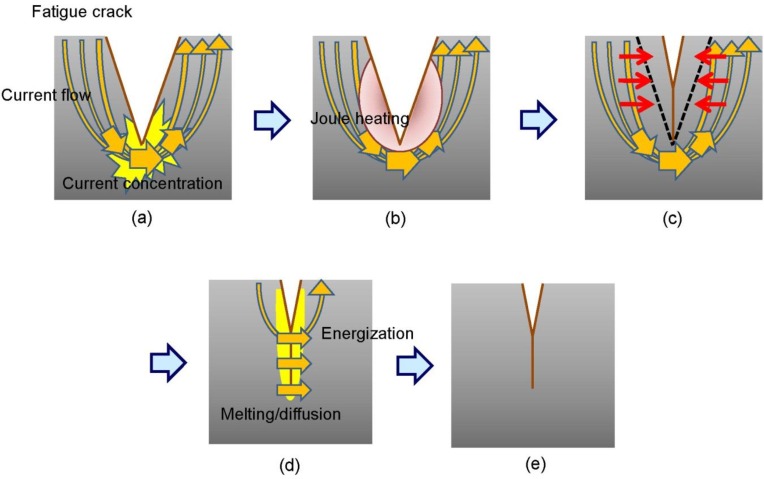
Representation of the crack closure process by the application of the electric current: (**a**) formation of a high-density electric current field at a crack tip; (**b**) local thermal expansion due to Joule heating; (**c**) crack closure resulting from thermal compressive stress; (**d**) bonding due to energization between the crack surfaces and (**e**) crack healing after the application of electropulsing.

**Figure 12:**
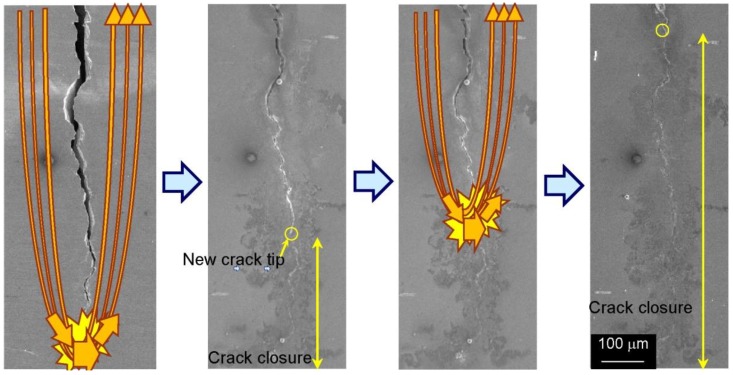
Closure of entire fatigue crack by multiple applications of high-density electropulsing.

One of the reasons why the crack closes and heals, as shown in Figure 5 and Figure 6, is thought to be the thermal compressive stress due to Joule heating as a result of the high-density electric current field formed at the crack tip [29]. The principle of the fatigue crack closing upon high-density electropulsing is represented schematically in Figure 11. When the electric current is applied across a crack, it flows along the crack because of the electric resistance on the crack surface. Therefore, a high-density electric current field is formed at the crack tip (Figure 11a). The area at the tip and in the vicinity of the crack is heated rapidly and expands because of Joule heating (Figure 11b). Conversely, the area away from the crack tip where the high-density electric field is not induced remains intact. Therefore, expansion is restricted and thermal compressive stress is caused, which brings about closure (Figure 11c). The crack surfaces then come in contact with each other and energization between the crack surfaces causes them to bond (Figure 11d). Moreover, it is thought that the crack surfaces are easily bonded because the oxide layer that otherwise prevents bonding is eliminated and the Ni film functions as an adhesive inner layer [28]. Finally, the crack remains closed after treating high-density electropulsing (Figure 11e), and the crack tip transfers in the direction of the notch. Therefore, the position of the current concentration continuously transfers in the direction of the notch and the whole crack closes. Figure 12 is a schematic representation of the fatigue crack being closed as a result of high-density electropulsing.

### 4.2. Fatigue Crack Growth Rate

The rate of crack growth in Sample B increased immediately after the application of high-density electropulsing as shown in Figure 9. We believe that the residual tensile stress at the crack tip caused the acceleration of crack propagation. Residual tensile stress can still be prevalent after the release of thermal stress. Once the extra thermal compressive stress is applied and the material yields at the crack tip, plastic deformation occurs at the crack tip. The thermal compressive stress is released when the current is stopped. However, the strain around the crack tip is restricted by the unheated elastic area. Therefore, we propose that residual tensile stress was prevalent in the area of crack closure. In contrast, the rate of crack growth in Sample C decreased immediately after the application of high-density electropulsing as shown in Figure 10. The delay in crack propagation was likely caused by the bridging and bonding between the crack surfaces. The difference in the crack growth rate immediately after the application of electropulsing between Samples B and C is attributable to the influence of the SAPC. In Sample C, the bonding between the crack surfaces is strong as a result of being treated with the SAPC, whereas in the untreated Sample B the bonding is weak. Thus, the effect of residual tensile stress is suppressed by the strong bonding between the crack surfaces in Sample C.

## 5. Conclusions

Healing due to the application of a controlled, high-density electric current was studied in fatigue cracks on surfaces passivated toward oxidation by the SAPC. Crack closure and bonding between crack surfaces were realized using this approach. Crack closure of 79%–89% was achieved as a result of the improved adhesion between crack surfaces after multiple applications of high-density electropulsing. Moreover, it was observed that the rate of crack growth decreases after the application of electropulsing. Therefore, this technique has the great potential to heal fatigue cracks in a range of metallic structures.
